# Immunophenotypes associated with bipolar disorder and lithium treatment

**DOI:** 10.1038/s41598-019-53745-7

**Published:** 2019-11-25

**Authors:** Tai-Na Wu, Chau-Shoun Lee, Bo-Jian Wu, Hsiao-Ju Sun, Chieh-Hsing Chang, Chun-Ying Chen, Chih-Ken Chen, Lawrence Shih-Hsin Wu, Andrew Tai-Ann Cheng

**Affiliations:** 1 Institute of Biomedical Sciences, Academia Sinica, Taipei, Taiwan; 2 Department of Medicine, MacKay Medical College; Department of Psychiatry, Mackay Memorial Hospital, Taipei, Taiwan; 3 Yuli hospital, Ministry of Health and Welfare, Hualien, Taiwan; 4 Tsao-Tun Psychiatric Center, Ministry of Health and Welfare, Nantou, Taiwan; 5 Bali Psychiatric Center, Ministry of Health and Welfare, Tamsui, Taiwan; 6 School of Medicine, Chang Gung University; Community Medicine Research Center & Department of Psychiatry, Chang Gung Memorial Hospital, Keelung, Taiwan; 7 Graduate Institute of Biomedical Sciences, China Medical University, Taichung, Taiwan; 8 Department of Psychiatry, China Medical University Hospital, Taichung, Taiwan

**Keywords:** Genetics, Immunology

## Abstract

Immune dysfunction is implicated in the etiology of bipolar disorder. The single-nucleotide polymorphism rs17026688 in the gene encoding glutamate decarboxylase–like protein 1 (*GADL1*) has been found to be associated with lithium response in Han Chinese patients with bipolar I disorder (BDI). However, whether patients with *GADL1* polymorphisms have different immunophenotypes is unknown. To address this issue, differences in the immune profiles based on analysis of peripheral blood mononuclear cells (PBMCs) were compared among BDI patients and healthy controls who lack or carry the T allele of rs17026688. BDI patients had significantly higher percentages of total T cells, CD4^+^ T cells, activated B cells, and monocytes than healthy controls, suggesting that immunologic imbalance might be involved in BDI development or progression. Treatment of BDI patients-derived PBMCs with lithium *in vitro* increased the percentage of CD14^+^ monocytes and dendritic cells, suggesting that lithium plays an immunomodulatory role in CD14^+^ monocytes and dendritic cells. Among BDI patients, non-T carriers had a significantly higher percentage of CD11b^+^/CD33^lo^/HLA-DR^−^ myeloid-derived suppressor cells than T carriers. Moreover, only T carriers exhibited differential sensitivity to lithium therapeutic use with respect to the percentage of myeloid cells. These findings suggest that rs17026688 polymorphisms in *GADL1* are associated with immune dysfunction in BDI patients.

## Introduction

Immune dysfunction is implicated in the etiology of bipolar disorder^1^. For example, changes in circulating leukocytes may contribute to the immunologic imbalance observed in patients with bipolar disorder^2,3^. Myeloid-derived suppressor cells (MDSCs) are a heterogeneous population of myeloid cells. Most human MDSCs express CD11b and CD33 and rarely express HLA-DR (MHC class II)^4^. MDSCs can potently suppress lymphocyte function, thereby posing a significant hurdle to anti-tumor immunity^4^. Production of arginase I (ARG1) is one of the most frequently reported mechanisms used by MDSCs to suppress T-cell functions^5,6^. ARG1 catalyzes the conversion of the amino acid L-arginine (L-Arg) to ornithine and urea. T cells activated under low L-Arg conditions fail to proliferate and express fewer T-cell receptors^7,8^.

Tumor-infiltrating regulatory T cells (Tregs) have been found to inhibit antitumor responses, and their rate of infiltration correlates with tumor progression^9^. In the majority of cancer patients, peripheral CD4^+^/CD25^+^/FOXP3^+^ and CD8^+^/CD28^−^ Tregs inhibit proliferative and cytotoxic T-cell responses^10^. CD8^+^/CD103^+^ Tregs also potently suppress T-cell responses^11,12^. When patients with bipolar disorder are compared with healthy controls, increased percentages of activated T (i.e., CD3^+^/CD25^+^ and CD3^+^/CD71^+^) and B (CD19^+^) cells^3^ as well as a lower percentage of CD4^+^/CD25^+^/FOXP3^+^ Tregs but a higher percentage of CD8^+^/CD28^−^ Tregs^2^ have been documented in different studies. However, studies with different subject inclusion/exclusion criteria or with patients having differing mood states (euthymic, manic or depressive at the time of blood collection) yielded discordant results^2,13^. Thus, the role of lymphocytes in the clinical course of bipolar disorder remains controversial.

Lithium has been a first-line choice for maintenance therapy of bipolar disorder and reduces the risk of relapse and suicide^14,15^. However, in studies of patients of European descent who were treated with lithium, only 30% had an excellent response with complete remission of symptoms^16,17^. Besides being a mood stabilizer for patients with bipolar disorder, lithium has been shown to increase the production of Th2 cytokines (e.g., IL-4 and IL-10) and decrease that of Th1 cytokines (e.g., IFNγ and IL-2) in an *ex vivo* assay of whole-blood cultures^18^. Blood monocytes have an altered proinflammatory status in patients with bipolar disorder, and lithium treatment might affect that status^19,20^.

Glutamate decarboxylase–like protein 1 (GADL1) has aspartate decarboxylase and cysteine sulfinic acid decarboxylase activities, catalyzing decarboxylation of aspartate, cysteine sulfinic acid, and cysteic acid to produce β-alanine, hypotaurine, and taurine^21^. Chronic lithium administration decreases taurine levels in the rat brain^22,23^. The enzyme activity of GADL1 increases in the presence of 0.2–0.4 mM lithium^24^. The single-nucleotide polymorphism rs17026688 in *GADL1* has been shown to be associated with lithium response in bipolar I disorder (BDI) patients of Han Chinese descent. Patients carrying allele T (CT and TT) at rs17026688 have a much better response to lithium treatment than those carrying the homozygous allele C^25,26^, although this association has yet to be replicated in other populations^27,28^. The variant in intron 8 of *GADL1*, IVS8 + 48delG, which is in complete linkage disequilibrium with rs17026688, is able to affect the splicing of *GADL1* mRNA^25^.

Based on these reports, we hypothesized that GADL1 modulates the effects of lithium on certain immunophenotypes of BDI patients. Therefore, we explored the immunophenotypes—including lymphocytes and myeloid cells—among BDI patients having different genotypes for *GADL1* rs17026688.

## Results

### Lymphocyte subsets between T and non-T carriers of rs17026688 among BDI patients and healthy controls

Table 1 presents the demographic and clinical characteristics of BDI patients and healthy controls. The characterization of total T, CD4^+^ T, CD8^+^ T, CD19^+^ B, CD56^+^/CD3^−^ natural killer (NK), and Treg (including CD4^+^/CD25^+^/FOXP3^+^, CD8^+^/CD28^−^, CD8^+^/CD103^+^) cells revealed no significant differences for their percentage distributions in the peripheral blood between T and non-T carriers among BDI patients or healthy controls. Only the percentage of CD56^+^/CD3^+^ natural killer T (NKT) cells differed significantly between T and non-T carriers among healthy controls. BDI patients had significantly higher percentages of total T and CD4^+^ T cells than healthy controls. Healthy controls had a significantly higher percentage of NK cells than BDI patients (Table 2).

**Table 1:** Demographic and clinical characteristics of bipolar I patients and healthy controls with rs17026688 polymorphisms.

	BDI vs. HC			BDI patients			HC		
	BDI	HC	p value	T carriers	non-T carriers	p value	T carriers	non-T carriers	p value
Group size	76	60		38	38		31	29	
Age, years (mean ± SD)	48.01 ± 9.88	31.68 ± 5.88	<0.0001***	49.08 ± 9.61	46.95 ± 10.16	0.35	31.74 ± 6.96	31.62 ± 4.56	0.94
Gender			0.026*			0.82			0.2
Men	31 (41%)	36 (60%)		15 (39%)	16 (42%)		21 (68%)	15 (52%)	
Women	45 (59%)	24 (40%)		23 (61%)	22 (58%)		10 (32%)	14 (48%)	
Psychotropic medications									
Lithium				17 (45%)	14 (37%)				
Carbamazepine				5 (13%)	5 (13%)				
Valproate				22 (58%)	21 (55%)				
Antidepressants				3 (8%)	6 (16%)				
Antipsychotics				28 (74%)	27 (71%)				
Benzodiazepines				27 (71%)	30 (79%)				

The differences in age were calculated using the two-tailed student’s t test, whereas those in gender were calculated using the χ^2^ test (Pearson). T and non-T carriers were compared among BDI patients or among healthy controls (HC).

**Table 2:** Immunophenotyping of immune cell populations in bipolar I patients and healthy controls with rs17026688 polymorphisms.

Markers	Cell type	BDI vs. HC			BDI patients			HC		
		BDI (%)	HC (%)	p-value	T (%)	non-T (%)	p-value	T (%)	non-T (%)	p-value
Total CD3^+^	T	54.59 ± 14.48	51.14 ± 13.89	0.036*	56.85 ± 15.63	52.75 ± 13.18	0.101	49.85 ± 14.06	52.53 ± 13.82	0.225
CD3^+^ CD4^+^	Th	28.52 ± 9.33	22.94 ± 10.58	0.042*	29.55 ± 10.63	27.50 ± 7.83	0.381	22.32 ± 10.15	23.61 ± 11.17	0.455
CD3^+^ CD8^+^	Tc	19.69 ± 7.03	21.04 ± 6.74	0.206	20.27 ± 6.66	19.12 ± 7.44	0.194	21.27 ± 6.58	20.8 ± 7.02	0.423
CD3^−^ CD19^+^	B	9.94 ± 4.79	8.75 ± 4.02	0.103	9.85 ± 5.16	10.03 ± 4.47	0.289	7.99 ± 3.89	9.56 ± 4.06	0.052
CD3^−^ CD56^+^	NK	13.05 ± 8.80	17.28 ± 9.43	0.011*	13.11 ± 9.67	13.00 ± 7.95	0.44	17.67 ± 10.70	16.87 ± 8.01	0.488
CD3^+^ CD56^+^	NKT	2.86 ± 2.25	2.72 ± 2.51	0.248	3.06 ± 2.51	2.66 ± 1.97	0.316	2.25 ± 2.23	3.22 ± 2.73	0.036*
CD3^+^ CD4^+^ CD71^+^	activated Th	4.24 ± 2.53	3.57 ± 2.52	0.079	3.81 ± 2.10	4.67 ± 2.84	0.12	3.59 ± 2.22	3.54 ± 2.85	0.328
CD3^−^ CD19^+^ CD71^+^	activated B	29.90 ± 13.52	16.32 ± 9.69	<0.0001***	30.30 ± 13.69	29.50 ± 13.52	0.497	17.61 ± 8.67	14.94 ± 10.65	0.061
CD4^+^ CD25^+^ FOXP3^+^	Treg	3.68 ± 1.94	4.64 ± 2.75	0.218	3.41 ± 1.84	3.05 ± 1.60	0.243	4.30 ± 1.86	5.00 ± 3.45	0.497
CD8^+^ CD28^−^	CD8^+^ Treg	38.47 ± 13.50	32.79 ± 15.92	0.552	37.17 ± 12.56	42.20 ± 12.22	0.057	29.90 ± 13.74	35.88 ± 17.67	0.08
CD8^+^ CD103^+^	CD8^+^ Treg	2.17 ± 1.13	2.17 ± 1.08	0.356	2.07 ± 0.75	2.27 ± 1.42	0.373	2.14 ± 1.03	2.20 ± 1.14	0.473
CD11b^+^ CD14^+^ CD15^−^ CD33^hi^ HLA-DR^+^	monocytes	9.98 ± 5.16	8.33 ± 3.76	0.006**	11.14 ± 5.89	8.82 ± 4.07	0.056	8.06 ± 3.28	8.61 ± 4.26	0.361
CD11b^+^ CD14^−^ CD15^+^ CD33^lo^ HLA-DR^−^	MDSC	41.40 ± 21.35	46.04 ± 22.12	0.29	34.46 ± 20.79	48.33 ± 19.83	0.002**	46.83 ± 20.99	45.19 ± 23.61	0.444

Abbreviations: Th, helper T; Tc, cytotoxic T; NK, natural killer; NKT, natural killer T; Treg, regulatory T; and MDSC, myeloid-derived suppressor cells. Data represent the mean (%) ± S.D. The differences between HC and BDI patients were assessed for statistical significance and adjusted for gender and age using general linear regression (*p < 0.05; **p < 0.01; ***p < 0.001). The significance of differences between T and non-T carriers was determined with the Mann-Whitney test (*p < 0.05; **p < 0.01). The percentages of CD4^+^ T, CD8^+^ T, CD19^+^ B, CD56^+^/CD3^−^ NK, and CD56^+^/CD3^+^ NKT cells were analyzed in the gated lymphocytes. The percentage of CD71^+^ cells was analyzed in the gated CD4^+^/CD3^+^ cells or CD19^+^ B cells. The percentage of CD25^+^/FOXP3^+^ Treg refers to gated CD4^+^/CD3^+^ cells, and the percentages of CD28^−^ and CD103^+^ cells were analyzed in the gated CD8^+^/CD3^+^ cells.

The activation status of CD4^+^ T and CD19^+^ B cells was examined using the proliferation marker CD71, which revealed no significant difference in the percentage of CD71^+^ cells in the CD4^+^ T or B cells between T and non-T carriers among BDI patients or healthy controls. BDI patients had a significantly higher percentage of CD71^+^ B cells than healthy controls (Table 2).

### Higher percentage of MDSCs in non-T than in T carriers among BDI patients

**Figure 1:**
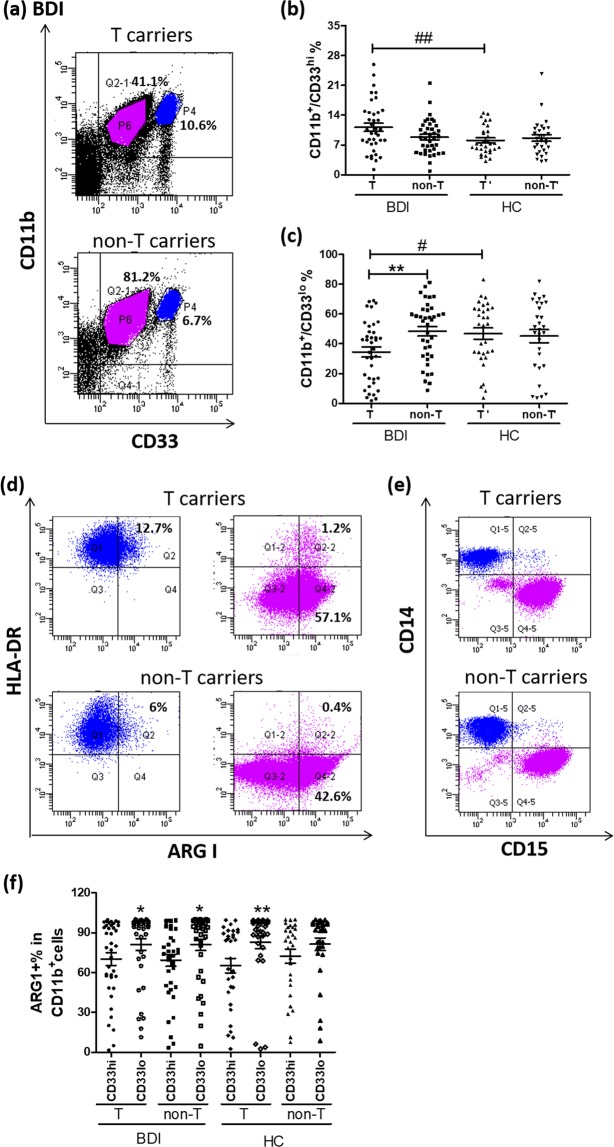
Characterization of CD11b^+^ cells with flow cytometry. PBMCs were stained with antibodies against CD11b, CD33, and HLA-DR on ice for 30 min. After fixation and permeabilization, cells were incubated with an antibody against ARG1 on ice for another 30 min. (**a**) Stained cells were analyzed using a flow cytometer, which revealed two distinct populations of CD11b^+^/CD33^hi^ (P4 gate) and CD11b^+^/CD33^lo^ (P6 gate) cells from BDI patients. The percentages of (**b**) CD11b^+^/CD33^hi^ and (**c**) CD11b^+^/CD33^lo^ cells between BDI patients and healthy controls (HC) were calculated and adjusted for gender and age using general linear regression (#p < 0.05; ##p < 0.01). Among BDI patients, only the percentage of (**c**) CD11b^+^/CD33^lo^ cells differed significantly between rs17026688 T and non-T carriers, as assessed with the Mann-Whitney test (**p < 0.01). These two groups of CD11b^+^ cells were further gated to examine the expression of HLA-DR and ARG1 (**d**) or of CD14 and CD15 (**e**). (**f**) The percentage of ARG1^+^ cells was compared between CD11b^+^/CD33^hi^ and CD11b^+^/CD33^lo^ populations in rs17026688 T and non-T carriers among BDI patients and HC using the Mann-Whitney test (*p < 0.05; **p < 0.01). Horizontal lines denote the mean ± SEM.

CD11b^+^ cells were examined with antibodies against CD33, HLA-DR, and ARG1 and then analyzed by flow cytometry, which revealed two distinct populations of cells, namely CD11b^+^/CD33^hi^ (P4 gate) and CD11b^+^/CD33^lo^ (P6 gate) (Fig. 1a). Among BDI patients, the percentage of CD11b^+^/CD33^hi^ cells did not differ significantly between T and non-T carriers (Fig. 1b and Table 2), whereas non-T carriers had a significantly higher percentage of CD11b^+^/CD33^lo^ cells (Fig. 1c and Table 2). BDI patients had a significantly higher percentage of CD11b^+^/CD33^hi^ cells than healthy controls (Table 2). For BDI patients versus healthy controls, only the T carriers exhibited a statistically significant difference in the percentages of CD11b^+^/CD33^hi^ (Fig. 1b) and CD11b^+^/CD33^lo^ cells (Fig. 1c).

Further characterization of the CD11b^+^/CD33^hi^ cells from both BDI patients and healthy controls revealed that most of these cells were HLA-DR^+^/CD14^+^/CD15^−^ (Fig. 1d,e) and that most of the CD11b^+^/CD33^lo^ cells were HLA-DR^−^/CD14^−^/CD15^+^ (Fig. 1d,e). Moreover, the CD11b^+^/CD33^lo^ cells secreted significantly greater amounts of ARG1 than CD11b^+^/CD33^hi^ cells from BDI patients and the T carriers of healthy controls (Fig. 1d,f). These results suggested that CD14^+^/CD15^−^/CD11b^+^/CD33^hi^/HLA-DR^+^ and CD14^−^/CD15^+^/CD11b^+^/CD33^lo^/HLA-DR^−^ cells have different properties, i.e., the latter might be more immunosuppressive than the former in terms of the amount of ARG1 secreted. Of the CD11b^+^/CD33^hi^ or CD11b^+^/CD33^lo^ cells, there was no difference in ARG1 secretion between BDI patients and healthy controls.

### Effects of *in vitro* lithium treatment on monocytes and dendritic cells

**Figure 2:**
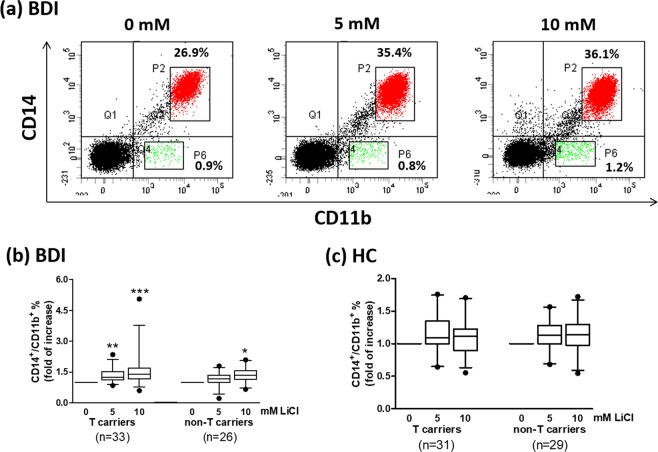
*In vitro* lithium treatment of BDI patient-derived PBMCs showed an increase in CD14^+^/CD11b^+^ monocytes but not CD14^−^/CD11b^+^ myeloid cells. PBMCs from rs17026688 T carriers (n = 33) vs. non-T carriers (n = 26) of BDI patients (**a,b**) or T carriers (n = 31) vs. non-T carriers (n = 29) of healthy controls (HC; c) were cultured with or without lithium for 6 days and then subjected to antibody staining for CD14 and CD11b on ice for 30 min. (**a**) The stained cells were analyzed using a flow cytometer, which revealed two distinct populations of CD14^+^/CD11b^+^ (P2 gate) and CD14^−^/CD11b^+^ (P6 gate) cells from BDI patients. *In vitro* lithium treatment of BDI patient-derived PBMCs showed a dose-dependent increase in CD14^+^/CD11b^+^ monocytes (**b**) but not CD14^−^/CD11b^+^ myeloid cells (**a**). The box plots show the median and quartiles, and the whisker caps of the box plots denote the mean 5th and 95th percentile values. The values measured at 5 and 10 mM LiCl were compared with values measured for nontreated cells using Tukey’s multiple comparison test (*p < 0.05; **p < 0.01; ***p < 0.001).

**Figure 3:**
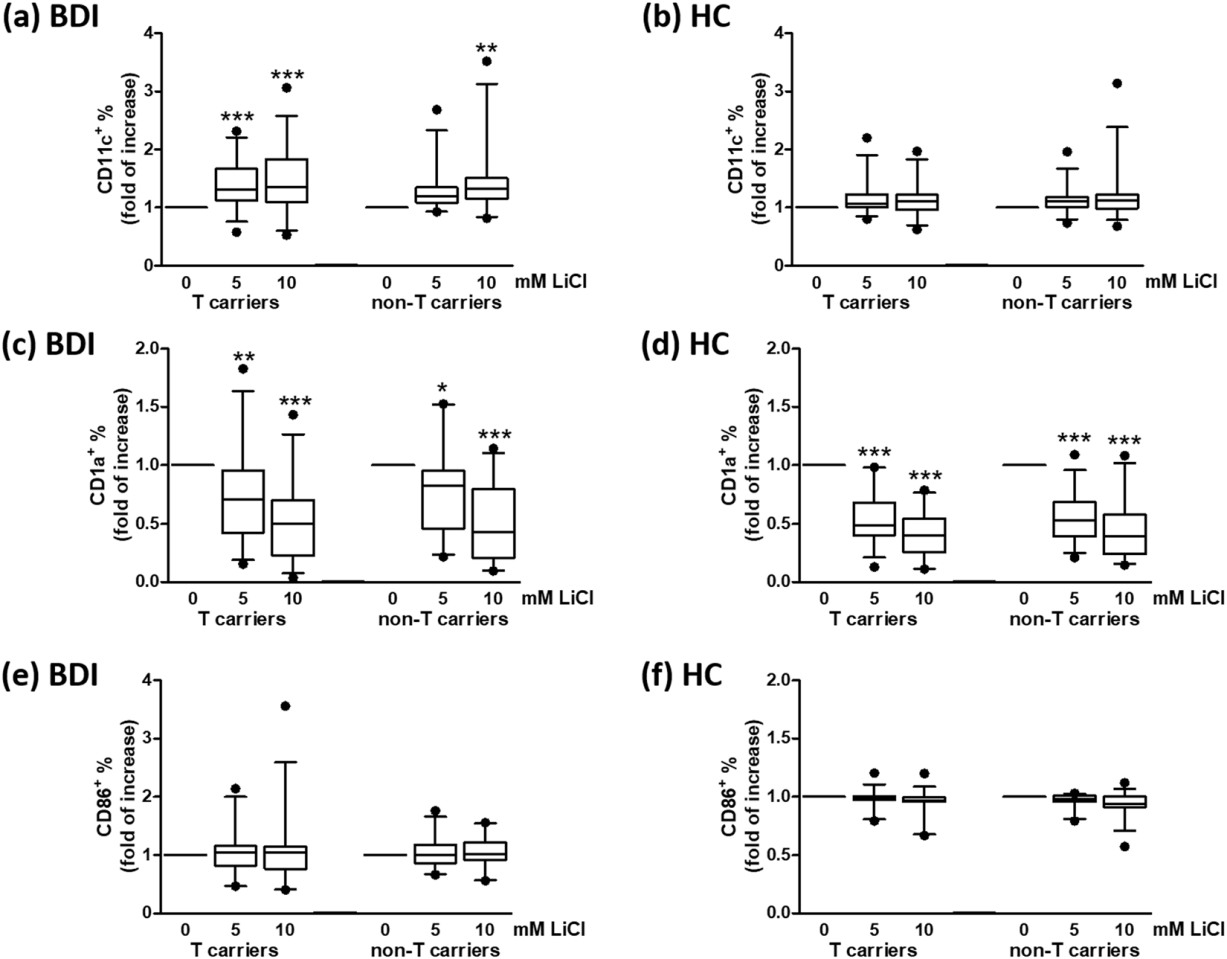
Treatment of PBMCs with lithium increases the percentage of CD11c^+^ dendritic cells and modulates their immune status in BDI patients. PBMCs from rs17026688 T carriers (n = 33) vs. non-T carriers (n = 26) of BDI patients (**a,c,e**) or T carriers (n = 31) vs. non-T carriers (n = 29) of healthy controls (HC) (**b,d,f**) were cultured with or without lithium for 6 days and then incubated with antibodies against CD11c, CD1a and CD86 on ice for 30 min. After washing, cells were subjected to fluorescence-activated cell sorting analysis. The CD11c^+^ cells (**a,b**) were gated to further analyze the expression of CD1a (**c,d**) and CD86 (**e,f**). The box plots show the median and quartiles, and the whisker caps of the box plots denote the mean 5th and 95th percentile values. The values measured at 5 and 10 mM LiCl were compared with values measured for nontreated cells using Tukey’s multiple comparison test (*p < 0.05; **p < 0.01; ***p < 0.001).

PBMCs were collected from BDI patients and then *in vitro* treated with different concentrations (0, 5, and 10 mM) of lithium for 6 days, showing that the percentage of CD14^+^/CD11b^+^ cells was increased, whereas the percentage of CD14^−^/CD11b^+^ cells did not change appreciably (Fig. 2a). No statistically significant differences were found between T and non-T carriers for PBMCs that were compared after being cultured with the same concentration of lithium. Addition of 5 or 10 mM lithium to PBMCs from T carriers resulted in an increase in the percentage of CD14^+^/CD11b^+^ cells (Fig. 2b) or CD11c^+^ dendritic cells (Fig. 3a). In comparison, treatment of PBMCs derived from non-T carriers only with 10 mM lithium could increase the percentage of CD14^+^/CD11b^+^ cells (Fig. 2b) or CD11c^+^ dendritic cells (Fig. 3a).

The treatment of BDI patient–derived PBMCs with lithium also decreased the expression of CD1a (MHC I–like antigen-presenting molecule; Fig. 3c) on dendritic cells, whereas the expression of CD86 (co-stimulatory molecule) was not affected (Fig. 3e).

For PBMCs from healthy controls, *in vitro* treatment with lithium did not significantly increase the percentage of CD14^+^/CD11b^+^ (Fig. 2c) or CD11c^+^ dendritic cells (Fig. 3b). The treatment of healthy control–derived PBMCs with lithium decreased the expression of CD1a (Fig. 3d) on dendritic cells, whereas the expression of CD86 did not change substantially (Fig. 3f). Taken together with the data presented in Fig. 1 and Table 2, these results revealed that rs17026688 polymorphisms have an effect mainly on myeloid cells in BDI patients.

### Effects of rs17026688 genotype and lithium treatment on immunophenotypes

**Figure 4:**
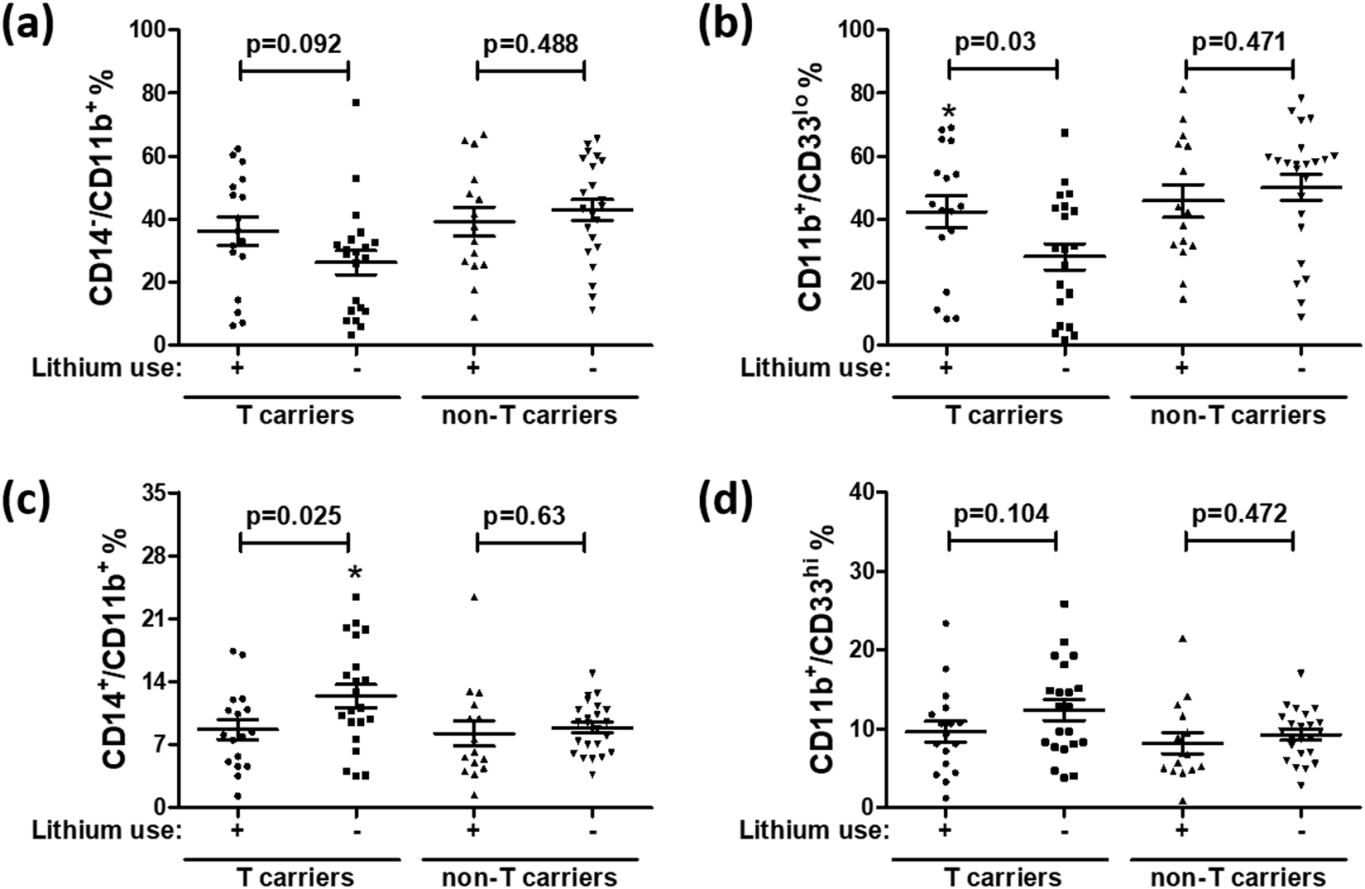
Effects of rs17026688 genotype and lithium therapeutic use on immunophenotypes of BDI patients. The percentages of (**a**) CD14^−^/CD11b^+^, (**b**) CD11b^+^/CD33^lo^, (**c**) CD14^+^/CD11b^+^, and (**d**) CD11b^+^/CD33^hi^ cells were compared between subgroups treated with or without lithium among T and non-T carriers of BDI patients (*p < 0.05). Using multiple regression analysis, the differences between subgroups treated with or without lithium were assessed for statistical significance and adjusted for the influence from other drugs (e.g., antidepressants, antipsychotics, and benzodiazepines). Horizontal lines denote the mean ± SEM.

Figure 4 shows effects of genotype (rs17026688) and the administration of lithium to BDI patients on the immunophenotypes of PBMCs. When assessing the effects of lithium therapeutic treatment, we used multiple regression analysis to adjust for the possible influence from other drugs, e.g., antidepressants, antipsychotics, and benzodiazepines.

Among T carriers of BDI patients, the percentage of CD11b^+^/CD33^lo^ (Fig. 4b) cells was higher in the lithium-treated group than in the nontreated group (p = 0.03). In comparison, the percentage of CD14^+^/CD11b^+^ (Fig. 4c) cells was higher in the nontreated group than in the lithium-treated group among the T carriers (p = 0.025). Moreover, among T carriers of BDI patients, lithium therapeutic use did not significantly alter the percentage of CD14^−^/CD11b^**+**^ (Fig. 4a) or CD11b^+^/CD33^hi^ (Fig. 4d) cells. Among non-T carriers of BDI patients, lithium therapeutic treatment did not significantly affect the immunophenotypes of all myeloid cells examined (Fig. 4a–d).

Our data indicate that, among BDI patients, the immunophenotypes of myeloid lineages of T carriers are sensitive to lithium therapeutic use, but this is not the case for non-T carriers. Taken together with the data presented in Fig. 1, these results indicate that rs17026688 polymorphisms affect the percentages of myeloid cells in peripheral blood as well as their sensitivity to lithium therapeutic treatment in BDI patients.

## Discussion

In this study, we report that BDI patients have significantly higher percentages of total T, CD4^+^ T, and CD71^+^ B cells than healthy controls, which echoes previous findings that patients with bipolar disorder have greater numbers of activated T and B cells^3^. On the other hand, BDI patients had a significantly lower percentage of NK cells than healthy controls. These findings suggest that changes in the frequencies of circulating lymphocytes may contribute to the immunologic imbalance observed in patients with bipolar disorder.

Myeloid cells may play a role in the development of bipolar disorder. In our study, BDI patients had a significantly higher percentage of CD14^+^/CD11b^+^ cells than healthy controls, which is consistent with published findings that monocytes from patients with bipolar disorder have altered expression of the monocyte marker CD14 and decreased ability to differentiate into fully active dendritic cells^20^. Together, these results indicate that changes in the percentage or phenotypes of circulating myeloid cells also contribute to the immunologic imbalance observed in patients with bipolar disorder.

Treatment of BDI patient–derived PBMCs with lithium *in vitro* increased the percentages of CD14^+^/CD11b^+^ population and dendritic cells. Besides, lithium treatment altered the status of dendritic cells in PBMCs from both BDI patients and healthy controls. CD1a expression has been found to be upregulated when monocytes are treated with granulocyte-macrophage colony-stimulating factor (GM-CSF) plus interleukin 4 (IL-4) *in vitro* to induce differentiation into dendritic cells^29^. Our observation of decreased expression of CD1a in PBMCs from BDI patients and healthy controls indicates that *in vitro* lithium treatment might hinder dendritic-cell maturation, which is consistent with the report that *in vitro* exposure to lithium hampers the generation of fully functional dendritic cells from monocytes^20^. Together, these results suggest that lithium has an immunomodulatory effect on CD14^+^ monocytes and dendritic cells of BDI patients, which is echoed by the findings that lithium can regulate monocyte and dendritic-cell responses in patients with bipolar disorder^19,20^. In addition to monocytes and dendritic cells, lithium can also impede the tumor-induced expansion of MDSCs—and therefore tumor growth—in mice^30^. Treatment with lithium is associated with reduced overall cancer risk in patients with bipolar disorder among the Han Chinese population^31^. We found that, among BDI patients, T carriers expressed a significantly lower percentage of MDSCs than non-T carriers, demonstrating for the first time that MDSCs might play a role in patients with bipolar disorder among the Han Chinese population.

Because *in vitro* treatment of PBMCs with lithium had a much greater effect on CD14^+^/CD11b^+^ cells than CD14^−^/CD11b^+^ cells, these two groups of cells might have different properties in BDI patients. We found that rs17026688 T carriers had a significantly lower percentage of CD11b^+^/CD33^lo^ cells compared with non-T carriers, but no such difference was apparent for CD11b^+^/CD33^hi^ cells among BDI patients. Most of the CD11b^+^/CD33^hi^ and CD11b^+^/CD33^lo^ cells were HLA-DR^+^ and HLA-DR^−^, respectively. Several reports have described human MDSCs as CD33^+^HLA-DR^−/low^ and CD11b^+^. This type of MDSCs often suppresses immune responses via the production of ARG1 or reactive oxygen species to decrease the levels of L-Arg^4,32^. Indeed, we found that CD11b^+^/CD33^lo^/HLA-DR^−^ cells secreted greater amounts of ARG1 than CD11b^+^/CD33^hi^/HLA-DR^+^ cells from both BDI patients and the T carriers of healthy controls. Similar findings have also been observed in bladder cancer patients, in that only the CD11b^+^/CD33^lo^/HLA-DR^−^ population had immunosuppressive activity^32^. In our study, BDI patients had a significantly higher percentage of CD14^+^/CD15^−^/CD11b^+^/CD33^hi^/HLA-DR^+^ cells than healthy controls, which is consistent with the previous finding that common genetic variants located in the HLA locus contribute to the risk of schizophrenia and bipolar disorder^33^. The expression amounts of CD33 in the monocytes are associated with monocyte function and amyloid biology in patients with Alzheimer’s disease^34,35^, suggesting an important role for myeloid lineage cells in the development of neurological and psychiatric diseases.

GADL1 is an enzyme responsible for decarboxylation of aspartate, cysteine sulfinic acid, and cysteic acid to produce β-alanine, hypotaurine, and taurine^21^. Neutrophils and monocytes express high levels of myeloperoxidase, which catalyzes the formation of the potent oxidant, hypochlorous acid. Taurine scavenges hypochlorous acid to form the more stable and less toxic adduct, taurine chloramine^36,37^, which can inhibit IL-1β and IL-6 production by lipopolysaccharide-stimulated PBMCs^38^. MDSCs isolated from mice bearing metastatic mammary tumors selectively express proteins involved in the metabolism of taurine and hypotaurine^39^. In addition, human peripheral blood monocytes upregulate GADL1 expression when cocultured with adipocytes^40^. We also observed that non-T carriers secreted higher amounts of GADL1 and taurine in the plasma than T carriers of BDI patients^41^. Together, these results offer possible explanations for our observation that rs17026688 polymorphisms in *GADL1* affect the percentage of MDSCs in peripheral blood of BDI patients.

Our study has certain limitations. First, we cannot rule out the possible effects of concomitant administration of psychotropic drugs on the immunological endophenotypes we examined. Second, upon blood-sample collection, the participants were not assessed using mood rating scales, such as the Beck Depression Inventory, Hamilton Depression Rating Scale, or Young Mania Rating Scale, although the clinical phenotype was assessed using a cross-culturally validated Chinese version of the Schedules for Clinical Assessment in Neuropsychiatry (SCAN)^42^, supplemented by available medical records and reports from family members and psychiatrists. Possible influences of subclinical mood changes on the measurements in this study cannot be ruled out, as the measurements were not adjusted for this aspect.

In conclusion, we found a significantly higher percentage of MDSCs in non-T than T carriers among BDI patients. Among BDI patients, only the T carriers differed significantly from healthy controls with respect to the percentages of CD11b^+^/CD33^hi^ and CD11b^+^/CD33^lo^ cells. Moreover, only T carriers exhibited differential sensitivities to lithium therapeutic use on the percentages of myeloid cells. These results suggest that rs17026688 polymorphisms in *GADL1* influence myeloid cells in BDI patients with respect to their peripheral blood distribution and the sensitivity toward lithium therapeutic treatment. For the first time, our findings show that rs17026688 polymorphisms are involved in the modulation of immune function in patients with bipolar disorder. MDSCs might play an immunomodulatory role in BDI patients of the Han Chinese population.

## Methods

### Subjects

A total of 76 euthymic BDI patients without autoimmune diseases, drug abuse, or alcoholism were recruited from the departments of psychiatry in general hospitals and psychiatric institutions in Taiwan. A total of 60 healthy controls without autoimmune, infectious disease or any mental disorder were also recruited for comparison. Table 1 presents demographic and clinical characteristics for all participants. BDI was diagnosed according to DSM-IV criteria for BDI with recurrent episodes of mania with or without depressive episode(s). Patients with other psychoses or affective disorders were excluded.

The study was approved by the medical or research ethics committees of Chang Gung Medical Foundation, Macky Memorial Hospital, YuLi Hospital Ministry of Health and Welfare, Tsao-Tun Psychiatric Center Ministry of Health and Welfare, Bali Psychiatric Center Ministry of Health and Welfare, China Medical University and Hospital, and Academia Sinica. Informed consent was signed by enrolled patients and healthy controls. All experiments were performed in accordance with relevant guidelines and regulations.

### Immunophenotyping

Samples of peripheral blood were collected and prepared as previously described^41^. PBMCs were isolated using Ficoll-Paque (GE Healthcare, US) density gradient centrifugation. The freshly isolated PBMCs were washed with phosphate-buffered saline containing 0.05% sodium azide and then treated with Fc Block solution (Miltenyi Biotec, Germany) on ice for 20 min. Next, cells were stained at 4 °C for 30 min with combinations of antibodies against CD3, CD4, CD8, CD19, CD14, CD25, CD28, CD33, CD11b, CD56, CD71, and CD103 (all from Biolegend, US). After surface staining, cells were washed and analyzed by fluorescence-activated cell sorting (FACSCanto II flow cytometer, BD Biosciences, US). For intracellular staining, cells were fixed and permeabilized (ebioscience, US) and then incubated with antibodies against FOXP3 (ebioscience, US) or ARG1 (R & D, US).

### *In vitro* PBMC culture

PBMCs from each subject were cultured for 6 days in RPMI 1640 medium supplemented with 10% fetal bovine serum, 2 mM l-glutamine, 100 U/ml penicillin, and 100 μg/ml streptomycin (all from Life Technologies, US) in the presence of 0, 5, or 10 mM LiCl (Sigma, US). Cells were then incubated with specific antibodies and subjected to fluorescence-activated cell sorting (FACS) analysis. Addition of 20 mM lithium to cells was found to yield an intracellular lithium concentration of 3.2 ± 0.2 mM as measured by a previous study^43^. Moreover, treatment of SH-SY5Y neuroblastoma cells with 20 mM lithium does not result in cytotoxicity in a previous report^44^.

### Statistical analysis

The immunophenotypic differences between HC and BDI patients were assessed for statistical significance and adjusted for gender and age using general linear regression. The Mann-Whitney test was used to assess differences in immune cell populations between T and non-T carriers at rs17026688. When the effects of lithium therapeutic treatment were assessed, multiple regression analysis was used to adjust for the possible influence from other drugs, e.g., antidepressants, antipsychotics, and benzodiazepines. Data were analyzed with SPSS software (version 19.0, Armonk, NY: IBM Corp.). All statistical tests were considered significant at the p < 0.05 level. GraphPad Prism 5 software was used for graphing data distributions.
